# Quality of life after primary antireflux surgery: an analysis by primary indication

**DOI:** 10.1007/s00464-025-11819-w

**Published:** 2025-05-27

**Authors:** Mark Shacker, Andrés R. Latorre-Rodríguez, Austin Reynolds, Sai Pidathala, Andrew Keogan, Sumeet K. Mittal

**Affiliations:** 1 Creighton University School of Medicine, Phoenix Health Sciences Campus, Phoenix, AZ USA; 2 Norton Thoracic Institute, St. Joseph’s Hospital and Medical Center, 500 W Thomas Road, Phoenix, AZ 85013 USA; 3 Grupo de Investigación Clínica, Escuela de Medicina y Ciencias de la Salud, Universidad del Rosario, Bogotá, D.C. Colombia

**Keywords:** Gastroesophageal reflux, GERD, Quality of life, Antireflux surgery, Fundoplication, GERD-HRQL

## Abstract

**Background:**

Antireflux surgery (ARS) includes all procedures performed at the hiatus, even those for indications other than typical gastroesophageal reflux disease (GERD). However, postoperative quality of life (QoL) across different indications remains poorly explored. We explored one-year QoL outcomes using the GERD-Health-Related Quality of Life (HRQL) instrument across four common indications and further described the trends over 5 years.

**Methods:**

After IRB approval, we retrospectively queried data from a prospectively maintained database of patients who underwent elective minimally invasive ARS by a single surgeon (November 2017–August 2023). Patients were included if they completed both a preoperative and 1-year postoperative GERD-HRQL questionnaire. Exclusion criteria were revisional surgeries, non-fundoplication procedures, emergencies, and lung transplantation. We analyzed one-year GERD-HRQL total and subcomponent scores (heartburn, dysphagia, regurgitation) and patient satisfaction by surgical indication: (i) volume reflux/typical GERD, (ii) obstructive symptoms/dysphagia, (iii) chronic bleeding/anemia, and (iv) atypical GERD. Further, QoL trends and acid suppression therapy use were analyzed over five years among the entire cohort.

**Results:**

A total of 83 patients were included. The proportion of eligible patients at 1, 2, 3, and 5 years was 83/83 (100%), 31/55 (56.4%), 24/43 (55.8%), and 16/26 (61.5%), respectively. The median total GERD-HRQL score of the entire cohort improved from 27 (IQR: 12.5–40.5) preoperatively to 0 (IQR: 0–4.5), 2 (IQR: 0–10.5), 3.5 (IQR: 2–12), and 1 (IQR: 0.75–4.5) at 1, 2, 3, and 5 years, respectively (all p < 0.05). At one year in the atypical GERD group, HRQL scores trended higher (i.e., worse QoL) and satisfaction trended lower.

**Conclusion:**

Although ARS improves QoL and patient satisfaction over time, current practice and research lack a stratified approach based on surgical indications and patient needs. Defining clear patient phenotypes and establishing specific surgical and patient-centered outcomes for each group should be the next priority.

**Supplementary Information:**

The online version contains supplementary material available at 10.1007/s00464-025-11819-w.

Antireflux surgery (ARS) is the gold standard for treating complex gastroesophageal reflux disease (GERD), offering excellent long-term outcomes [1]. Primary outcome measures for this procedure have primarily focused on objective metrics such as disease recurrence, severity, and the need for acid suppression therapy. More recently patient-reported outcome measures (PROMs), including quality of life (QoL) and patient satisfaction, are being increasingly reported [2–4].

Over the last two decades, the importance of PROMs has slowly been recognized within the surgical community. Quality of life metrics directly reflect patient satisfaction and serve as an indirect measure and possibly a more relevant measure of procedural success, as patients may report poor QoL despite normal objective findings and vice versa [5, 6]. Additionally, although subjective by nature, the sequential assessment of QoL provides valuable insights into the patient's overall well-being, allowing surgeons to track and evaluate patient progress more comprehensively after surgery. As a result, several validated instruments, such as the GERD-Health-Related Quality of Life (GERD-HRQL) tool, have been developed to capture and quantify this outcome.^5^ However, despite the widespread adoption of the GERD-HRQL questionnaire, few studies have examined the long-term (i.e., ≥ 5 years) outcomes in QoL improvements after ARS, leaving the available evidence on this topic somewhat scant and unclear [7–10].

Furthermore, metrics that quantify pre- and postoperative QoL differ according to the patient’s chief complaint or surgical indication—an area that has not received much attention. ARS is a broad umbrella term that includes all procedures performed at the hiatus including those for typical GERD symptoms (e.g., heartburn, volume reflux) as well as those for atypical symptoms (e.g., chest pain or chronic cough) or hiatal hernia (HH)-related conditions (e.g., chronic anemia, incarceration, obstructive symptoms). However, outcomes data—both clinical and patient-centered—are mixed and poorly defined for indications beyond volume reflux/typical GERD [11–13].

We hypothesize that ARS provides long-term improvements in both QoL and patient satisfaction, though variations may exist depending on the primary surgical indication. Thus, we aimed to explore how QoL and patient satisfaction differ at one-year after surgery based on four common indications for ARS as well as to describe these PROM trends over a five-year period.

## Methods

### Study design, setting and ethics

We conducted an observational, retrospective, cohort study to evaluate QoL and patient satisfaction after primary elective ARS. Data were queried from a prospectively maintained database containing demographic and surgical information as well as pre- and postoperative results of QoL assessments of patients who underwent minimally invasive benign foregut procedures by a single experienced surgeon (S.K.M.) between November 2017 and August 2023 at a tertiary care center in the U.S.A. This study was approved under the Norton Thoracic Institute Foregut Umbrella Protocol by the Institutional Review Board for Human Research at St. Joseph’s Hospital and Medical Center in Phoenix, Arizona (PHXU-21-500-136-73-18, project approval date: 27–Nov-2023). Due to the study design, written patient consent was waived. The study adhered to the good practice guidelines of the Helsinki Declaration and followed the STrengthening the Reporting of OBservational Studies in Epidemiology (STROBE) statement and checklist (Supplementary Material S1).

### Study population

Patients aged 18 years or older were included if they underwent primary elective ARS (robotic or laparoscopic) during the study period and completed both the preoperative assessment and at least the first-year scheduled postoperative follow-up, including the GERD-HRQL questionnaire. Patients were excluded if they (i) underwent another antireflux procedure (e.g., magnetic sphincter augmentation, EndoStim, transoral incisionless fundoplication, or Roux-en-Y gastric reconstruction), (ii) required an emergency procedure, (iii) had a history of prior foregut procedures, or (iv) were a lung transplant candidate or recipient.

### Study outcomes

The primary outcomes included quality of life, assessed by the GERD-HRQL total score and subcomponent scores (heartburn, regurgitation, dysphagia), and overall patient satisfaction at one year based on the primary surgical indication for ARS. Additionally, a longitudinal analysis evaluated extended trends of these outcomes for up to five years, including all eligible patients regardless of surgical indication. Secondary outcomes included rates of acid suppression therapy use, specifically proton pump inhibitors (PPIs), at each follow-up.

### Perioperative care and patient follow-up

#### Preoperative workup

The preoperative assessment for patients with GERD and/or HH undergoing primary elective ARS at our institution has been previously described [14]. Briefly, all patients undergo an in-person evaluation, including a comprehensive medical history and physical examination by an experienced foregut surgeon. Esophageal function testing—such as barium esophagram, esophagogastroduodenoscopy, high-resolution manometry, and esophageal pH monitoring—is performed as needed. Based on these findings, surgical treatment is offered and tailored to appropriate candidates.

#### Antireflux surgery

ARS was performed using either a laparoscopic or robotic approach, following a standardized sequence of steps in all cases: (i) excision of the hernia sac, (ii) esophageal/mediastinal mobilization, (iii) high mediastinal dissection, (iv) cruroplasty—which, in cases of a large HH or intrathoracic stomach, could include the use of a bioabsorbable U-shaped mesh—and (v) fundoplication (partial Toupet fundoplication was the preferred technique in most cases).

#### Postoperative care and follow-ups

After surgery, patients are monitored in the post-anesthesia care unit and transferred to the inpatient floor. Diet is advanced to liquids before discharge, typically within 1–2 days in uncomplicated cases. The first postoperative follow-up visit is scheduled ~ 21 days after surgery, with a second visit at 3 months. Subsequent follow-ups occur annually on the surgery anniversary until year 3, then biennially. Starting at the 1-year follow-up, patients are invited to complete the GERD-HRQL questionnaire and rate their overall satisfaction.

### Primary surgical indications

For the primary analysis at one year, patients were categorized into four groups based on the primary indication for ARS determined by the operating surgeon: (i) volume reflux/typical GERD, (ii) obstructive symptoms such as dysphagia or postprandial distress, (iii) chronic bleeding/anemia, and (iv) atypical GERD symptoms. A patient may have indications falling into more than one category; however, only the most pressing/concerning indication was used to categorize patients into the above groups.

#### Volume reflux/typical GERD

Patients diagnosed with severe GERD, with or without a concomitant HH. GERD is confirmed by endoscopic findings (e.g., erosive esophagitis, Barrett's esophagus) and/or pH-monitoring results (e.g., pathological DeMeester score). These patients primarily present with symptoms such as heartburn and regurgitation.

#### Obstructive symptoms/dysphagia

Patients whose primary complaint is difficulty swallowing (i.e., dysphagia), postprandial distress such as shortness of breath, or inability to belch when uncomfortable. They often have a large hiatal or paraesophageal hernia with possible organo-axial volvulus noted on radiographic studies or endoscopy.

#### Chronic bleeding/anemia

Patients with a moderate to large hiatal hernia, potentially without clear gastrointestinal symptoms, who are referred for surgical evaluation due to evidence of Cameron ulcers, chronic anemia, a history of transfusions, or the need for iron infusions. These include patients in whom no other cause is identified even after an exhaustive and prolonged workup by a gastroenterologist and/or a hematologist.

#### Atypical GERD symptoms

Patients with objective evidence of GERD, as confirmed by pH-testing, and complaints of atypical GERD symptoms, such as chronic cough, chest pain, hoarseness, or asthma-like symptoms. These patients may lack classical symptoms of heartburn and regurgitation.

### Patient-reported outcome measures

Patient QoL is assessed preoperatively and at scheduled postoperative follow-ups using the modified GERD-HRQL questionnaire, a 15-item instrument evaluating three symptom domains: heartburn, regurgitation, and dysphagia [15]. The heartburn and regurgitation sections each contain six questions, with each response scored from 0 to 5, yielding a maximum of 30 points per section. The dysphagia section includes two questions, also scored from 0 to 5, with a maximum of 10 points. An additional question on medication use contributes five points, bringing the total possible score to 75, where 0 represents optimal disease-specific QoL and 75 indicates the worst possible QoL. Additionally, three separate and optional questions assess (i) overall patient satisfaction categorized as “Satisfied,” “Neutral,” or “Dissatisfied”; (ii) overall satisfaction quantified on a 0–10 scale, with 10 representing the best outcome; and (iii) whether the patient would recommend the procedure to a relative or friend, “Yes” or “No.” The questionnaire is administered in person during clinic visits, via a structured phone interview by the clinic nurse, or via a secure email survey using Research Electronic Data Capture (REDCap).

### Sampling and sample size

A maximum sample size was achieved through convenience sampling, including all available individuals who met the inclusion criteria.

### Statistical analysis

Descriptive statistics were applied as appropriate. Continuous variables are reported as median and interquartile range (IQR) and categorical variables as count and proportion. Distribution for continuous data was assessed using the Shapiro–Wilk test and Q–Q plots. Comparisons of primary outcomes at one year were performed based on the primary surgical indication. Fisher’s exact test and the Kruskal–Wallis test were used for between-group comparisons. Additionally, the Wilcoxon rank-sum and McNemar tests were used to assess differences in changes between pre- and post-operative PROMs (i.e., GERD-HRQL scores and patient satisfaction) as well as the use of PPIs at each follow-up. Missing data were handled with pairwise deletion (available-case analysis), and p-values were adjusted by Bonferroni correction for multiple comparisons. A significance (alpha) level was set at 0.05 and all analyses were conducted using R version 4.4.1 (Foundation for Statistical Computing, Vienna, Austria).

## Results

### Study population characteristics

**Fig. 1:**
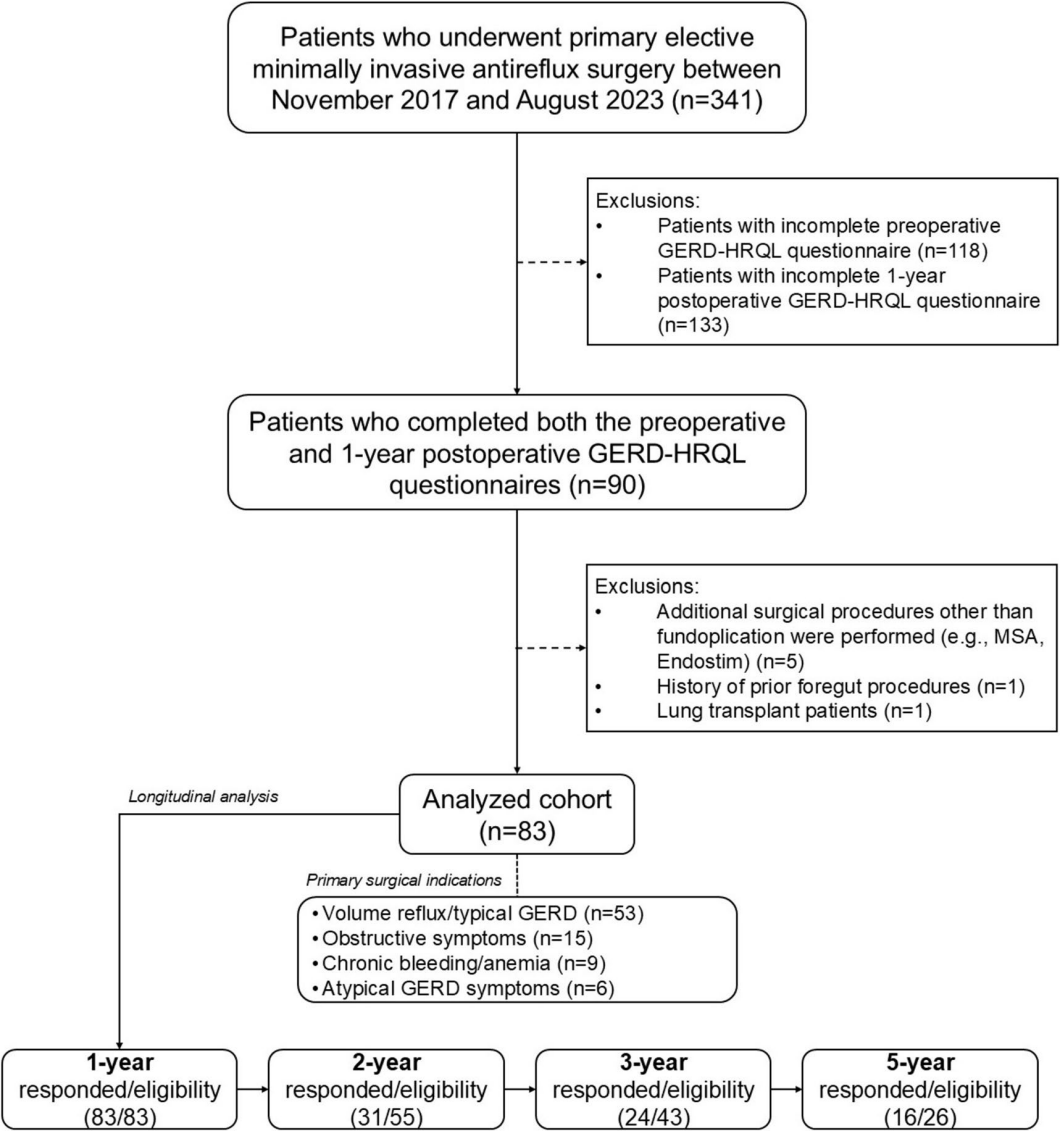
Study flow diagram. *GERD* gastroesophageal reflux disease, *HRQL* health-related quality of life, *MSA* magnetic sphincter augmentation

A total of 341 patients who underwent primary ARS during the study period were assessed for eligibility. Among them, 223 (65.4%) completed the preoperative GERD-HRQL questionnaire, and 233 (68.3%) completed it postoperatively at any time point. However, only 90 (26.4%) had completed both the preoperative questionnaire and the 1-year follow-up GERD-HRQL. Additional exclusions included patients who underwent antireflux procedures other than fundoplication (n = 5), had a history of prior foregut interventions (n = 1), or were lung transplant patients (n = 1). As a result, the analyzed cohort consisted of 83 patients. Among them, the proportion of eligible patients who completed all consecutive GERD-HRQL questionnaires at each postoperative follow-up was as follows: 83/83 (100%) at 1 year, 31/55 (56.4%) at 2 years, 24/43 (55.8%) at 3 years, and 16/26 (61.5%) at 5 years. The study flow diagram is presented in Fig. 1.

Most patients in the study cohort were female (n = 58 [69.9%]), the median age was 65 years (57–72), and the median BMI was 28.9 kg/m^2^ (26.3–31.7). The most common primary indication for ARS was volume reflux or typical GERD symptoms (n = 53 [63.9%]), followed by obstructive symptoms (n = 15 [18.1%]), bleeding or anemia (n = 9 [10.8%]), and atypical GERD symptoms (n = 6 [7.2%]). ARS was performed using a conventional laparoscopic approach in 71 (85.5%) patients, while 12 (14.5%) underwent robotic-assisted surgery. All patients underwent fundoplication: Toupet fundoplication, n = 73 (88%), Angle of His accentuation alone, n = 5 (6.0%), Dor fundoplication, n = 4 (4.8%), or Nissen fundoplication, n = 1 (1.2%). Additionally, cruroplasty with mesh reinforcement was performed in 23 cases (27.7%). Table 1 summarizes the baseline demographic and surgical characteristics of all patients as well as subgroup data based on the primary surgical indication.

**Table 1:** Baseline demographic and surgical characteristics of the cohort including stratification by primary indication for antireflux surgery

	Cohort N = 83	Atypical GERD n = 6	Bleeding or anemia n = 9	Obstructive symptoms n = 15	Volume reflux/typical GERD n = 53	p-value
Demographic characteristics						
Sex, female	58 (69.9)	4 (66.7)	6 (66.7)	11 (73.3)	37 (69.8)	1.000
Age, years	65 (57, 72)	61 (58, 68)	61 (57, 65)	76 (64, 77)	65 (55, 70)	.415
BMI, kg/m^2^	28.9 (26.3, 31.7)	29.8 (28.2, 31)	32.5 (27.6, 35.6)	28.4 (26.2, 31.5)	28.9 (26.3, 31.3)	1.000
Surgical and perioperative characteristics						
Surgical approach						
Laparoscopic	71 (85.5)	6 (100)	8 (88.9)	13 (86.7)	44 (83)	1.000
Robotic	12 (14.5)	0 (0)	1 (11.1)	2 (13.3)	9 (17)	
Operative time, min	85.0 (70, 110)	72.5 (60, 90)	100.0 (90, 120)	100.0 (80, 140)	80.0 (70, 100)	.341
Blood loss, mL	20.0 (10, 25)	7.5 (5, 50)	25.0 (15, 30)	25.0 (10, 50)	15.0 (10, 25)	.615
Intraoperative complications	5 (6)	1 (16.7)	0 (0)	0 (0)	4 (7.5)	1.000
Esophageal length, cm	2.0 (1.5, 2)	2.0 (2, 2.5)	1.5 (1.5, 2)	1.5 (1.5, 2)	2.0 (1.5, 2)	.481
LOS, days	1.0 (1, 2)	1.0 (1, 1)	1.0 (1, 1)	1.5 (1, 2)	1.0 (1, 1)	1.000
Mesh use	23 (27.7)	1 (16.7)	3 (33.3)	5 (33.3%)	14 (26.4)	1.000
Fundoplication type						
A.O.H accentuation	5 (6)	0 (0)	0 (0)	2 (13.3)	3 (5.7)	1.000
Dor fundoplication	4 (4.8)	0 (0)	0 (0)	3 (20)	1 (1.9)	
Nissen fundoplication	1 (1.2)	0 (0)	0 (0)	0 (0)	1 (1.9)	
Toupet fundoplication	73 (88)	6 (100)	9 (100)	10 (66.7)	48 (90.6)	
Percent intrathoracic stomach, %	25.0 (10, 66)	15.0 (7.5, 57.5)	40.0 (25, 50)	75.0 (50, 100)	22.5 (10, 40)	**.032**
Postoperative complications (Clavien–Dindo > 1)	4 (4.8)	0 (0)	1 (11.1)	2 (13.3)	1 (1.9)	1.000

All data presented as no. (%) or median (Q1, Q3). Bold indicates statistical significance, p < 0.05. Between groups comparisons were assessed using the Fisher’s exact test or Kruskal–Wallis test adjusted by the Bonferroni method for multiple comparisons

*A.O.H* angle of His, *BMI* body mass index, *GERD* gastroesophageal reflux disease, *LOS* length of hospital stay

### 1-year patient-reported outcome measures by primary surgical indication

Patients with an indication of volume reflux/typical GERD symptoms had the highest median preoperative total GERD-HRQL scores, though not statistically different from other groups (atypical GERD: 20 [15.2–36], bleeding or anemia: 9 [6–11], obstructive symptoms: 24 [17.5–44], volume reflux/typical GERD: 32 [18–43]; p = 0.153). Similarly, the severity of symptoms evaluated by the GERD-HRQL tool before surgery did not clearly differ between surgical indications, although as expected, patients with volume reflux/typical GERD trended toward higher heartburn subscores than the other groups (atypical GERD: 8.5 [6.2–17.5], bleeding or anemia: 2 [0–5], obstructive symptoms: 13 [8–25.5], volume reflux/typical GERD: 15 [10–21]; p = 0.153).

Furthermore, 76 patients (91.6%) experienced QoL improvement at 1 year after ARS, and the total and subcomponent GERD-HRQL scores were significantly lower at 1 year compared to preoperative values across all indications (p < 0.01). Notably, no statistical difference in the severity of any symptom captured by the GERD-HRQL was noted one year after surgery based on the primary surgical indication. However, atypical GERD patients trended toward higher postoperative GERD-HRQL scores compared to other indications, primarily due to relatively elevated regurgitation subscores. Table 2 summarizes preoperative and 1-year postoperative QoL assessments across the groups.

**Table 2:** Preoperative and 1-year postoperative GERD-HRQL total score and subscores by primary surgical indication

	Total GERD-HRQL score		Heartburn subscore		Regurgitation subscore		Dysphagia subscore	
	Pre-op	1 Year post-op	Pre-op	1 Year post-op	Pre-op	1 Year post-op	Pre-op	1 Year post-op
Indication								
Atypical GERD (n = 6)	20 (15.2, 36)	8 (1, 19.5)**	8.5 (6.2, 17.5)	1 (0, 8)**	9.5 (2.2, 11.5)	5.5 (0.5, 11.2)**	2 (1.2, 5)	0 (0, 2.2)**
Bleeding or Anemia (n = 9)	9 (6, 11)	0 (0, 1)**	2 (0, 5)	0 (0)**	1 (0, 7)	0 (0)**	1 (0, 1)	0 (0)**
Obstructive Symptoms (n = 15)	24 (17.5, 44)	0 (0)**	13 (8, 25.5)	0 (0)**	11 (3.5, 16.5)	0 (0)**	1 (0, 4.5)	0 (0)**
Volume Reflux (n = 53)	32 (18, 43)	1 (0, 6)**	15 (10, 21)	0 (0, 3)**	14 (2, 17)	0 (0)**	2 (0, 4)	0 (0, 1)**
Entire cohort (N = 83)	27 (12.5, 40.5)	0 (0, 4.5)**	13 (7, 21)	0 (0, 2)**	10 (1.5, 17)	0 (0)**	2 (0, 4)	0 (0, 0.5)**

Data reported as median (Q1, Q3). Scores at 1-year follow-up were compared to preoperative scores using the Wilcoxon rank-sum test adjusted by the Bonferroni method. *p-value < .05, **p-value < .01

*GERD* gastroesophageal reflux disease, *HRQL* health-related quality of life

We also investigated patient satisfaction. Preoperatively, patients with volume reflux/typical GERD, atypical GERD, and obstructive symptoms had relatively higher dissatisfaction rates, whereas those with bleeding or anemia reported lower dissatisfaction rates (atypical GERD: 83.3%, bleeding or anemia: 44.4%, obstructive symptoms: 80.0%, volume reflux/typical GERD: 81.1%). At 1 year after ARS, only 5 patients (6.0%) were completely dissatisfied with their condition, and the median satisfaction scores were as follows: 10 (9–10) for patients with typical GERD, 9.5 (7.25–10) for those with obstructive symptoms, 10 (9.5–10) for those with chronic bleeding/anemia, and 7 (6–9) for the atypical GERD group.

### Trends in quality of life and patient satisfaction (up to five years)

**Fig. 2:**
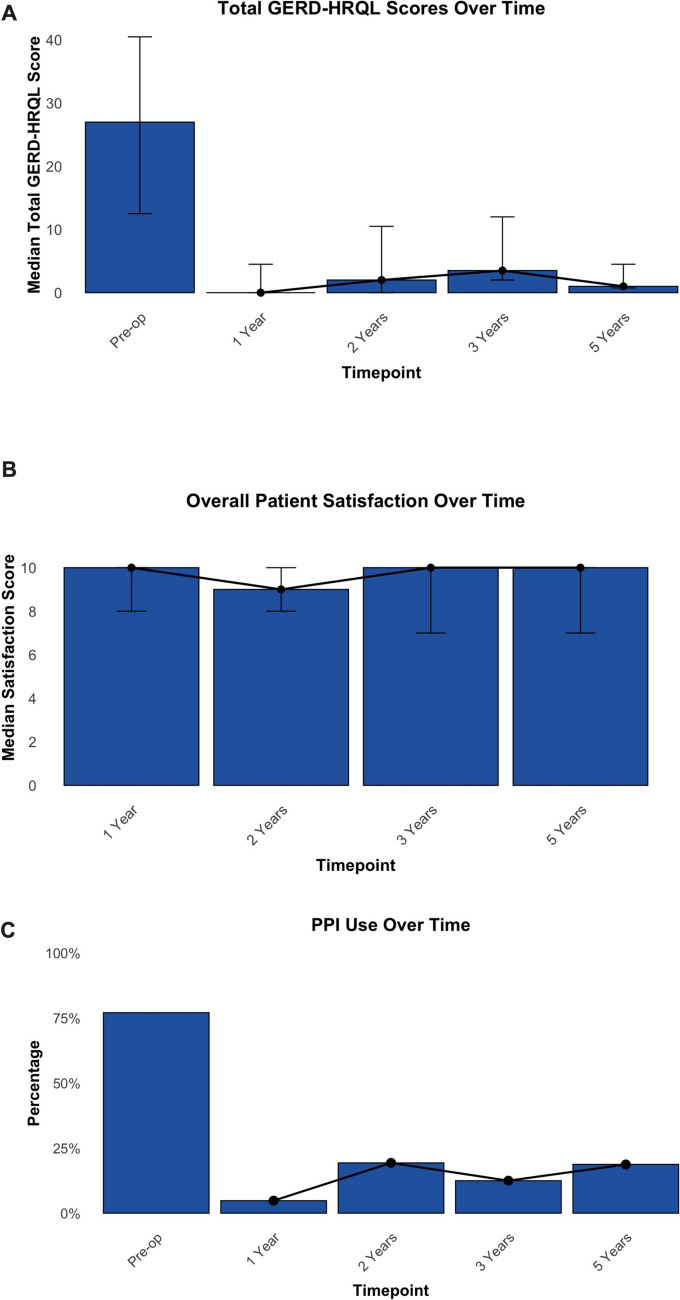
Follow-up of patients 1, 2, 3, and 5 years after ARS**. A** Changes in total GERD-HRQL scores over time on a scale of 0 to 75 (0 represents optimal disease-specific QoL and 75 indicates the worst possible QoL). **B** Overall patient satisfaction, assessed using a visual analog scale (0–10), with 10 indicating the highest level of satisfaction and** C** PPI use rates preoperatively and at 1, 2, 3, and 5 years postoperatively. Bars represent median values, with whiskers indicating the first and third quartiles. *GERD* gastroesophageal reflux disease, *HRQL* health-related quality of life, *PPI* proton pump inhibitor

We performed a longitudinal analysis of up to five years describing trends in the cohort’s QoL, satisfaction, and PPI use across the entire cohort. The median preoperative total GERD-HRQL score was 27 (12.5–40.5), with subscores for heartburn, regurgitation, and dysphagia of 13 (7–21), 10 (1.5–17), and 2 (0–4), respectively. Postoperatively, ARS resulted in a sustained improvement in QoL, with minimal recurrence of evaluated symptoms. The median total GERD-HRQL scores were 0 (0–4.5) at 1 year, 2 (0–10.5) at 2 years, 3.5 (2–12) at 3 years, and 1 (0.75–4.5) at 5 years (Fig. 2A). Similarly, median subcomponent scores remained significantly lower than preoperative values at all follow-up time points (p ≤ 0.01 for all comparisons). Table 3 summarizes the total and subcomponent GERD-HRQL scores preoperatively and at each scheduled postoperative follow-up.

**Table 3:** Pre- and postoperative GERD-HRQL total and subcomponent scores after antireflux surgery for the entire cohort (up to 5 years)

	Preoperative N = 83	1 Year N = 83	2 Years N = 31	3 Years N = 24	5 Years N = 16
Total score	27 (12.5, 40.5)	0 (0, 4.5)**	2 (0, 10.5)**	3.5 (2, 12)**	1 (0.75, 4.5)**
Heartburn subscore	13 (7, 21)	0 (0, 2)**	1 (0, 5.5)**	0 (0, 4.25)**	1 (0, 3.25)**
Regurgitation subscore	10 (1.5, 17)	0 (0, 0)**	0 (0, 0)**	0.5 (0, 4.5)**	0 (0, 1.5)**
Dysphagia subscore	2 (0, 4)	0 (0, 0.5)**	0 (0, 1)**	0 (0, 1.25)**	0 (0, 1)**

Data reported as median (Q1, Q3). Scores at each follow-up were compared to preoperative scores using the Wilcoxon rank-sum test adjusted by the Bonferroni method. * p-value: < .05, ** p-value: < .01

*GERD* gastroesophageal reflux disease, *HRQL* health-related quality of life

Patient satisfaction also improved after ARS. Preoperatively, 78 (94%) patients reported being either indifferent or dissatisfied with their medical condition. However, at all postoperative follow-ups, the proportion of patients who reported to be dissatisfied was notably lower (1-year: 6.0%, p < 0.001; 2-years: 9.7%, p = 0.013; 3-years: 21%, p = 0.209; 5-years: 6.3%, p = 0.384). Overall, median satisfaction with the procedure remained stable over time, reported as 10 (8–10) at 1 year, 9 (8–10) at 2 years, 10 (7.5–10) at 3 years, and 10 (7–10) at 5 years (Fig. 2B). Additionally, among the 72 patients who responded to the optional question, 63 (87.5%) indicated they would recommend the procedure to a relative or friend at any follow-up point.

### Trends in proton pump inhibitor use (up to five years)

Overall, 77.1% of patients reported PPI use before the antireflux procedure. A significantly lower proportion of patients reported use of any PPI at all subsequent postoperative follow-ups, although a slight trend towards increasing use over time was noted (1-year: 4.8%, p < 0.001; 2-years: 19.4%, p = 0.002; 3-years: 12.5%, p < 0.001; 5-years: 18.8%, p = 0.063; Fig. 2C). Unfortunately, the indication for the postoperative initiation of PPIs was not available.

## Discussion

Historically, the success of surgical interventions has been evaluated from the perspective of providers and healthcare systems, focusing on metrics such as morbidity, mortality, and costs. However, an increasing emphasis on patient-centered care has introduced PROMs as a critical component of the outcomes equation [16, 17]. The routine collection and analysis of PROMs after surgery not only provide insights into patients’ experiences and needs but also may allow more personalized postoperative care and refine surgical decision-making to improve long-term patient satisfaction and QoL [17]. Despite their growing adoption, long-term PROMs—including QoL and patient satisfaction—remain underexplored in foregut surgery.

As expected, in our longitudinal analysis of 83 patients who underwent primary minimally invasive ARS, we found an overall significant and sustained improvement in QoL among the cohort, as evidenced by reductions in total GERD-HRQL scores and subscores (i.e., heartburn, regurgitation, and dysphagia) and high patient satisfaction, persisting for up to five years postoperatively. Our findings align with those reported by other authors [5, 18, 19], and confirm that benefits derived from ARS extend beyond the usually evaluated clinical outcomes.

Indeed, the novelty of our findings may arise from the analysis conducted to evaluate differences in QoL one year after surgery among various patient phenotypes/surgical indications. Although the results demonstrated that QoL (as measured by the GERD-HRQL questionnaire) improved after ARS, regardless of the primary surgical indication—with only minimal variations—it is evident that the current approach to evaluating and reporting the "success" of the procedure, as well as monitoring patients, lacks sufficient personalization. ARS is performed for several indications beyond typical GERD, volume reflux, or symptomatic HH; however, patients are being evaluated and followed uniformly, resulting in a saturation of the medical literature reporting similar outcomes in mixed populations [20–22].

We believe that a paradigm shift is necessary to fully bring the concept of personalized medicine into the field of foregut surgery and more effectively tailor perioperative care to individual patients. To achieve this, it is essential to initiate a discussion among surgeons and professional societies to define clear patient categories (i.e., phenotypes) for those undergoing elective ARS. In our study, we have highlighted this need by categorizing the broad spectrum of patient presentations into four commonly accepted groups. We acknowledge that this approach may be insufficient as there is an overlap of symptoms between groups, and only the most pressing indication was used to categorize patients. Therefore, a future, well-designed, evidence-based or expert consensus may be required to refine this classification scheme (i.e., likely requiring a larger number of categories).

Furthermore, the lack of categorization by surgical indication may be just the tip of the iceberg. A deep view of this problem highlights that outcomes—whether surgical, cost-related, or PROMs—are not directly comparable, given the variability in the underlying surgical indications. This discrepancy raises concerns about the widespread use of unifaceted instruments such as the GERD-HRQL questionnaire alone. Although specifically designed and validated to assess QoL in patients with typical GERD [23], this instrument is often applied in clinical studies to populations with differing surgical indications [20–22]. Our study provides a clear example of this mistake. In other words, while the GERD-HRQL questionnaire may be routinely administered to all patients, it does not necessarily capture meaningful information for individuals whose primary complaint is different than typical GERD symptoms (e.g., chronic cough or anemia), nor does it accurately measure QoL in these different surgical scenarios.

Future approaches to personalized therapy should consider “hard” clinical outcomes and specific PROMs, using various surgical indications. For example, in patients with volume reflux, pH monitoring has been classically used to quantify GERD severity; however, results may not fully reflect the patient’s clinical experience when compared to GERD-HRQL scores. Similarly, patients undergoing surgery for chronic anemia or bleeding are often assessed based on postoperative hemoglobin improvement; a potential PROM in this group is the resolution of anemia-specific treatment and symptoms. Patients with obstructive symptoms, typically evaluated through barium esophagram or endoscopy for hernia recurrence, may require the use of a validated dysphagia-specific instrument. Of note, a subset of these patients presents with incarceration symptoms, and perhaps these patients could be further subdivided, and an outcome measure for them would be the absence of postprandial distress. For those with laryngopharyngeal or respiratory symptoms secondary to reflux, validated questionnaires such as the Hull Airway Reflux Questionnaire and Reflux Symptom Index may be used to supplement objective findings [24, 25]. These instruments could be used alongside objective measures to improve outcome evaluation and patient-centered care in the diverse group of patients who undergo ARS.

Our study has several limitations. Patient inclusion was determined by the completion of paired preoperative and first-year postoperative GERD-HRQL questionnaires. These inclusion criteria limited the sample size, as many patients who attended later follow-up visits but not the first-year postoperative visit were not included. The criteria may also have introduced selection bias, as patients who recovered well postoperatively may have been more likely to be lost to follow-up, whereas those who returned may have been more prone to present with refractory symptoms. Although, less likely given the overall follow-up rates, dissatisfied patients may have sought care elsewhere, which may have contributed to attrition bias. Further, the relatively small sample size and heterogeneity within each surgical indication group may limit the robustness of the subanalysis; however, statistical techniques were applied to mitigate the potential increase in Type I error. As previously discussed, our study relied on the GERD-HRQL questionnaire to assess QoL and patient satisfaction; while this is a validated tool, its applicability is limited to typical GERD symptoms. Additionally, despite following patients for up to five years, it was not possible to make reliable comparisons beyond 2 years between the groups due to the relatively high attrition rates; however, the benefits of ARS are likely to persist beyond this period, and extending the observation window would provide further insights. Finally, the generalizability of our results is limited, as the data reflect the practice of an experienced foregut surgeon at a high-volume center.

## Conclusion

Overall, primary minimally invasive ARS is associated with significant and sustained improvements in quality of life, high patient satisfaction rates, and low use of acid suppression therapy for most patients up to five years postoperatively. However, while patient-centered outcomes are essential in tracking non-clinical aspects of surgical success over time, current practice and research lack a stratified approach tailored to specific surgical indications and individual patient needs. Therefore, the next priority in the foregut surgery research agenda should be to define clear phenotypes of patients undergoing ARS, establish targeted surgical outcomes, and develop and/or validate instruments to measure these outcomes across each group.

## Supplementary Information

Below is the link to the electronic supplementary material.

Supplementary file1 (DOCX 30 KB)

## Data Availability

The data analyzed in this study is allocated in a REDCap database and it can be queried for future studies; however, it cannot be shared outside of those authorized as research staff per protocol. Access to this dataset requires IRB approval; if needed, direct to the corresponding author.
